# Hydrophobia and Imagination

**Published:** 1886-08

**Authors:** W. H. Forwood

**Affiliations:** Major a rgeon, U. S. Army; 505 La Salle Avenue


HYDROPHOBIA AND IMAGINATION. *By* W. H. FORWOOD, M. D., *Major a[illegible]rgeon, U. S. Army.*

On a beautiful moonlight evening, September 10th, 1868, the military post of Fort Larned, Kansas, was suddenly invaded by a mad wolf. The post was situated in those days in the midst of a vast rolling prairie far from any human habitation, save the encampments of roving Indian tribes, and on the plains in all directions were great bands of buffalo, wolves and other wild animals.

The night was clear and warm, and about 10 o'clock the patients in the ward of the little one-story adobe hospital were startled by the entrance of a large gray wolf. Corporal McGillicuddy, 3d Infantry, in hospital for sore eyes, rose up in bed, thinking to scare the animal off, but he was instantly attacked and severely bitten in the left hand and right arm, when the wolf rushed out through the open door and disappeared as suddenly as he came.

A moment later he was at a tent near by where two women were sleeping, and tore a large hole in the side of the canvas, leaving the marks of his teeth on the bed rail, without doing further damage.

His next appearance was at my own quarters, where he ascended the porch, bounded through the open window, tearing a large piece out of the curtain, sprang over the lounge on which I was lying at the time, and encountered my large Newoundland dog "Prince," in the middle of the room. The conflict that ensued was short and sharp; a furious rush, a few vicious snaps of his powerful jaws, and the wolf was off through the hall and out at the back door, apparently satisfied with the mischief he had done without any desire to fight the battle to a finish. His course from this point was a bee line across an open space of about 100 yards to the house of the Indian agent, where a number of persons were assembled, enjoying the beautiful evening on the veranda.

Lieutenant Thompson, 3d Infantry, was seated on the steps and saw the wolf approach. He came at a run, and without hesitating seized the officer, first by one leg and then the other, inflicting quickly several severe bites, and was gone in a moment at the same rapid pace in the direction of the cavalry stables.

On a cot in the open air in front of the stables, Private Mason, of the 10th Cavalry, was lying with his shoes and stockings off, awaiting his turn to go on post as one of the guard. The wolf seized him by the bare foot and drove his enormous canine tooth entirely through between the metatarsal bones from the instep to the sole.

The sentinel walking post across the way had not sufficient time to bring his gun to bear before the infuriated animal was upon him, and fired over its back as it plunged headlong between his legs, throwing him to the ground, and disappeared toward the hay stacks, leaving no more serious damage than an ugly rent in the leg of the soldier's pantaloons.

The guard at the hay stacks saw the wolf coming in time and making a lucky shot sent a bullet through his chest into the spinal column, killing him instantly. He was a large male specimen of his kind, very numerous at that time on the surrounding prairies. His mouth was covered with a frothy saliva, tinged with blood, traces of which he had left on every object touched in his entire round of the post. Indians relate similar instances of mad wolves entering their camps, and say they always remain and continue their depredations as this one did until killed.

The wounds of the three men bitten were promptly examined and all thoroughly washed in warm water and cauterized with the nitrate of silver. Lieutenant Thompson had numerous scars on both legs from the ankles to the knees, inflicted *through the ordinary clothing.* Private Mason's foot bled freely and was soaked for some time in several changes of water and then cauterized as deeply as possible from both openings of the wounds. Corporal McGillicuddy had the flesh stripped from his left little finger, leaving the bone almost bare. In the right arm the bites were deep, and except near the wrist were made through the covering of his cotton night shirt. I urged the immediate amputation of his finger at the metacarpo-phalangeal articulation, but he positively refused to give his consent. I suggested hydrophobia, but he laughed at the idea, saying wolves never went mad, and that there was no danger. It was not until the second or third day after the accident that he would consent to the amputation, when ether was given and the operation done. No attempt was made to treat the wounds of the dog, as his heavy coat of shaggy hair made it difficult to find them.

All the wounds, including the amputation, healed quickly and kindly. Lieutenant Thompson and Private Mason remained free from any unpleasant symptoms and are still living, although left to their own reflections, as they were for many months in that dreary and monotonous place, far from home and friends and fully cognizant of the fate that befell the dog and the other man, and told many stories by the Indians of the fatal effects of such accidents, they could not have been entirely free from anxiety.

Poor old Prince remained well and happy as usual until about the tenth day, when for the first time in years he showed signs of serious illness. He was dull, mopy, did not care to go out and refused all food or drink. When water was placed before him he appeared to want it, but would make no effort to swallow. He was securely chained in a comfortable shady place on the grass and carefully attended, but did not improve. On the afternoon of the second day he seemed very nervous, restless and unnatural in disposition and still refused to eat or drink. At this stage of the case an officer whose anxiety for the safety of his children was too great to tolerate any further experiments with a mad dog in the vicinity of his quarters, put an end to all further observation by shooting him through the head; but there was no doubt left in my mind as to the nature of the disease.

McGillicuddy was an old soldier, and I managed to keep him pottering about the hospital on the pretext of a slight granulation of the lids. He was otherwise perfectly well and cheerful, and showed no trace of fear or anxiety concerning his wounds. He knew that Thompson and Mason were well and on duty, and the fate of Prince was carefully kept from him. He had been on the frontier for many years and scouted the idea of a mad wolf as something too absurd to mention.

On the morning of the thirty-first day after the accident, in making my rounds of the ward I found the corporal lying on his bed. He did not get up and stand " at attention " as was his pride and custom, answered my questions in monosyllables, and was evidently out of sorts. At nine o'clock that evening the steward sent word that McGillicuddy was acting strangely and seemed quite ill. He was nervous, restless, cross, and wore an anxious expression. He complained of a smothering or choking sensation about the chest and throat, which he attributed to the *ether* administered when his finger was amputated, and scolded me roundly for having made him take it. I noticed every now and then a peculiar spasmodic action of the diaphragm—a succession of two or three quick, short, jerking movements—giving rise to a sort of sob or sigh, such as may be noticed in young children at the end of a long fit of crying. This occurred once in two or three minutes when quiet or oftener when disturbed and was quite characteristic. Some little simple sedative mixture was prepared and offered him in a glass, but he discovered to his surprise that he could not take it, nor even think of doing so without great distress. After a little persuasion he took the glass, holding it as far from him as possible, and in great agitation he assured me he could not swallow. Finally, after several attempts, he succeeded in bringing the glass with a trembling hand almost to his lips, then throwing it violently from him fell back in convulsive excitement, sighing rapidly and deeply as above described. From this time the man never swallowed a particle of anything until just before the close, when the attendants thought they had succeeded in getting a few drops of water down his throat from a teaspoon. This abstinence was not from any obstinacy on the part of the patient, but because swallowing was impossible and the attempt distressed and excited him more than almost anything else.

The spasmodic disturbance beginning in the muscles of the diaphragm and throat extended in the next twenty-four hours to the chest, back and extremities, and the nervous sensitiveness increased until it became extreme. The least disturbance, the slightest noise, the gentlest touch sufficed to bring on spasmodic excitement. So far as the muscular system was concerned these convulsive attacks bore a striking resemblance to those caused by poisonous doses of strychnine. Every fibre of the muscles seemed powerfully contracted for a time, then slowly relaxed, and the patient, whose mind remained clear throughout, would beg those about him to keep still and make no noise, that the intervals of comparative rest might be a little longer. The effort to talk distressed him, and he therefore spoke as little as possible, sometimes complaining of thirst and a burning sensation in the throat and mouth and of the frightful pains that accompanied his attacks. Death closed the scene at the end of seventy hours after the first noticeable symptoms appeared. Not the slightest sign of fear was exhibited at any time, and almost the last word the patient uttered was a complaint against the doctor for giving him ether, which he believed to be the main cause of his illness.

Much has been written of late by prominent men in the medical profession tending to show that true rabies in man is a myth, the so-called cases of hydrophobia being merely the result of fear and imagination. It seems to me that a sufficient refutation of this theory is the fact that rabies is communicated, through the bite, from one animal of the canine species to another, and from these to beasts of other species, as horses, cows, pigs, sheep, etc. It will hardly be claimed that fear or imagination plays any important part in determining the result in such cases as these, which are numerous and well authenticated; and if thus communicable to the lower animals, why not to man also? It is certain that the disease originating in dogs and wolves and characterized by the definite and well recognized symptoms long known under the name of hydrophobia is no myth but a painful and invariably fatal reality. Precisely the same symptoms are transmissible from one dog to another through the bite, and similar symptoms to other animals, each according to its species. Those produced in man are characteristic and distinguishable from all other known diseases. In order to a clear understanding of the matter it is necessary to bear in mind the following important points:

1st. Rabies in the canine species, though probably liable to epidemic outbreaks, is quite rare, but many other diseases of dogs have been mistaken for it, thus giving rise to numerous spurious cases and much unnecessary alarm.

2nd. The bite of a rapid dog does not always, nor even in a majority of cases, infect the animal bitten, just as vaccination does not always "take" in children, although the virus may be good. It may not be introduced into the blood or may be washed off before it has been absorbed, or possibly the system may be from some unknown cause protected against its effects. Of a given number of animals bitten all remain well for a longer or shorter period, and then a small proportion sicken and die and the rest show no ill effects other than those resulting from the slight wounds inflicted, which are no more serious than if made by a healthy dog. But, once the symptoms of hydrophobia appear, death is inevitable. There is no middle ground. Recovery, so far as any well authenticated instance to the contrary is concerned, is *prima facie* evidence of non-infection and erroneous diagnosis. PREVENTION by means of washing, cauterization, etc., of the wounds is the only effectual treatment.

Imagination plays an important rôle in medical pathology and fear is a serious complication in many diseases, and it is to be expected that among intelligent beings, fully conscious of all the horrible consequences liable to follow the bite of a rabid animal, there should be more or less nervous disturbance and mental agony which in certain persons may lead to serious or even fatal results; but this will not hold good in the case of babies, idiots, insane persons or any of the lower animals, quite as liable to hydrophobia as the most imaginative men or women in the full possession of their mental faculties.

505 La Salle Avenue.

